# Reflex Irritation from the Rectum as a Cause of Some Affections of the Uterus

**Published:** 1869-06

**Authors:** J. C. Nott

**Affiliations:** New York


### REFLEX IRRITATION FROM THE RECTUM AS A CAUSE OF SOME AFFECTIONS OF THE UTERUS.

By J. C. NOTT, M.D., New York.

(Read before the New York Obstetrical Society.)

That dysmenorrhœa, amenorrhœa, menorrhagia, irritable uterus, engorgement of this organ, versions, flexions, prolapse, etc., are occasional results of reflex irritation, emanating from the rectum, will be conceded by the experienced practitioner; but my observation would lead me to believe that such cases are far more common than the profession is generally willing to acknowledge.

I have seen many examples of this kind, and am just now concluding the treatment of two typical cases, which have been under my care for three months. The cases are of equal interest, and either one would serve well to illustrate the positions I shall take; but I select that of Mrs. C., for the reason that Dr. J. Marion Sims saw it with me several times, was much interested in its history, and can vouch for the correctness of my statements.

The subject was a lady from the South, moving in the best society, aged thirty-seven, fair skinned, blue eyes, very handsome and intelligent, the mother of six children, and had lost her husband two years ago. She was robust, her labors had been very easy, and she never had any illness until after an abortion at three months, which occurred about three years ago. I should except hemorrhoids, which had been more or less an annoyance to her ever since the birth of her first child, fourteen years ago.

On the third day after the abortion, she started on a journey of one hundred miles, and was near flooding to death on the way. She apparently recovered well from this, and became robust again, having no trouble about the uterus until within the last twelve months, during which time her hemorrhoids have become worse, and during the same time she has complained a good deal of dysmenorrhœa.

During the summer of 1868, her symptoms gradually became more aggravated, and when I was called to see her about 1st September, she was confined to her room, and had not been out of the house for two months. The symptoms presented to me on full examination were as follows:—

*Nervous system* much deranged; neuralgic pains in back, in and around pelvis, face, and neck; no appearance of injection in coats of eye, but photophobia and inability to read; dyspeptic and without appetite; inability to sleep much, principally from pelvic pains.

The *rectum* presented, on examination, three external and two internal hemorrhoids, but no ulcer or fissure was discovered. There was, however, morbid contraction of the sphincter, and the introduction of the finger was very painful, and defecation was excessively so, both during and for an hour or two after the act. She complained also of excessive constipation, and never had a passage without an enema or purgative; but the subsequent history proves that what she termed constipation was the result of the morbid condition of the rectum and spasm of sphincter. The fæces sometimes collected for several days, became very hard and impacted, and doubtless had some agency in producing the displacement of uterus described below. The hemorrhoids bled but little, and her condition was the reverse of anœmia. The tumors all protruded during defecation, and often produced much suffering.

The *uterus* was engorged, everywhere tender to the touch, particularly the fundus, and was strongly anteflexed. She had been suffering for the last twelve months with dysmenorrhœa, and at times very severely. Though flexed, I found the neck abundantly open. By drawing a little upon the os with a tenaculum, so as to straighten the bend, I passed a large sound in so easily as to lead me to believe that the dysmenorrhœa was caused by spasm of internal os, originating in reflex irritation from the rectum. The menstruation was but little disturbed in its quantity, quality, and periodicity. There was no leucorrhœa worth speaking of, but a slight erosion around the os uteri. The sound passed in about three inches, confirming the hypertrophy indicated by the touch.

The left *ovarium* had long been a prominent point of pain, and could be easily felt by bimanual palpation. It was extremely sensitive to the touch, and about double its normal size. It seemed to be so prominent a point of complaint, and our means for combating such affections so unsatisfactory, that Dr. Sims hinted strongly at the experiment of electrolysis.

All attempts at walking aggravated her symptoms, and it seemed that every nerve in and around the pelvis was in a super-sensitive state—pressure, even on the external surface of pubic bones, sometimes could not be borne. Having repeatedly seen similar trains of symptoms caused by diseases of the rectum, I determined, in the first place, to clear the hemorrhoids out of the way and then stop, take an observation, and see what tack should next be taken. Another reason for beginning here, was the fact that it was really the most prominent point of suffering.

I accordingly removed the external tumors with scissors and ligatured the internal ones. Being exceedingly nervous, she suffered a good deal for thirty-six hours, and I was obliged to give opiates. The ligatures came away in five days, and the case progressed satisfactorily. The first evacuation on the third day was painful, but after this her so-called constipation disappeared, her discharges passed off freely and easily, and she was delighted at being relieved from her accustomed suffering.

As the case was likely to be tedious, and the lady was anxious to return as soon as possible to the South, I proposed a consultation with Dr. Marion Sims, hoping he could aid me in bringing the case to a speedy close. The rectum being relieved, we thought the next indication was the relief of the anteflexion; and I must state in this connection, what I have inadvertently omitted, *viz.*, that she suffered greatly from irritability of the bladder; she frequently had to get up eight or ten times during the night to urinate, and the urine was loaded with mucus (these symptoms I thought at the time were dependent solely on the pressure of the fundus of uterus upon the bladder, but this I now doubt, as both the bladder and uterus symptoms disappeared with irritation of the rectum.) We attempted to rectify the flexion by pessaries—Hodge's closed lever, Graily Hewitt's flexed lever, and the globe pessary were tried in turn, but so sensitive were the parts that we were compelled to abandon them all.

I then, with the hope at least of palliation of pains, fell back on another expedient which answered marvellously well, relieved all the disagreeable sensations, and enabled her at once to get up and take exercise with comfort. The following was the expedient alluded to—a soft, round sponge, about the size of a pullet's egg, was saturated with a solution of morphine and sulphite of soda and inserted into the vagina (℞ sulph. morphine, grs. v.; sulphite soda, ℥ss.; aqua, O j.)

This was worn through the night and directed to be renewed night and morning. When I called about noon the next day, I was delighted, and I may add surprised, to find her walking about the house in high spirits, after a good night's sleep, the first she had had for some time, and entirely free from pain.

The sponge prepared in the same way, and tepid vaginal washes of salt and water, were continued for a month, during which time she had good nights, the bladder symptoms, the tenderness of the ovarium, the innumerable pelvic pains, the photophobia, the dyspepsia and loss of appetite all disappeared —she not only walked up and down stairs freely, but was out walking in the streets every day through this city and gained strength daily. At the end of a month the sponge was discontinued, the use of salt and water continued, and she has now for ten days more continued well.

It is worthy of especial remark that she has menstruated three times since the hemorrhoids were removed and without the slightest pain, showing clearly, I think, that the dysmenorrhea did not depend on the flexion, but upon the engorgement and spasmodic contraction of the internal os, caused by reflex irritation from the rectum.

The engorgement of the uterus has greatly diminished, but the flexion continues and will probably be permanent.

There is another point in the case of practical interest, that I would beg to fix attention on. She thought, as I did, that the rectum disease was permanently relieved by the removal of the hemorrhoids; but a month after the operation, and a few days before I commenced the use of medicated sponge, she commenced complaining smartly of her old constipation and pain in defecation. On examination I found a small unhealed spot at the seat of one of the piles that had been removed by ligature, and producing all the characteristic symptoms of fissure of the anus. I gave her chloroform, inserted my thumbs into the anus, and so forcibly dilated the sphincter as to feel its fibres distinctly break. Two days after she had a stool without pain and has had none since, now a month.

This is an occurrence I have noticed repeatedly, and particularly in irritable, nervous females. This little ulcer forms at the seat of the pile, produces all the symptoms of fissure, and is relieved by the same treatment.

Now I repeat that this case may be regarded as a typical one of a large class, and I think we may refer all the complications to reflex action emanating from the rectum.

The moral I draw from the above is this: Never treat uterine affections without inquiring minutely into the condition of the rectum. Hemorrhoids, small ulcers, irritability, spasmodic contraction of sphincter, etc., are common causes of affections of the uterus, as well as vaginismus and other affections of the vagina; coccyodynia (a short paper on which I have before presented to the Society, and which appeared in the *American*

*Journal of Obstetrics* for November, 1868) is another fertile source of reflex irritation.

One word with regard to the use of the morphine and sulphite of soda. The objects in using the sulphite were two—First, it had gained some reputation as an application to inflamed surfaces; second, it is an antiseptic which would prevent the sponge from becoming foul. The indication for the morphine is clear enough, and although this application relieved the sensitive surface and nerves, is it at all probable that relief would have followed, had not the rectum—the fountain of irritation—been relieved? I deemed it important also to use the softest sponge that could be obtained.—*Am. Jour. of Obstetrics.*

